# Supplementary material for: A rapid and affordable point-of-care test for detection of SARS-CoV-2-specific antibodies based on hemagglutination and Artificial Intelligence-based image interpretation
Source: Res Sq. 2021 Jul 20:rs.3.rs-712902. Preprint. [Version 1] doi: 10.21203/rs.3.rs-712902/v1 (PMC8312898; doi:10.21203/rs.3.rs-712902/v1)
Supplement: Supplement 3 [file 7104a0016beb6b5fc8a792d1.pdf]

Supplementary Figure 1

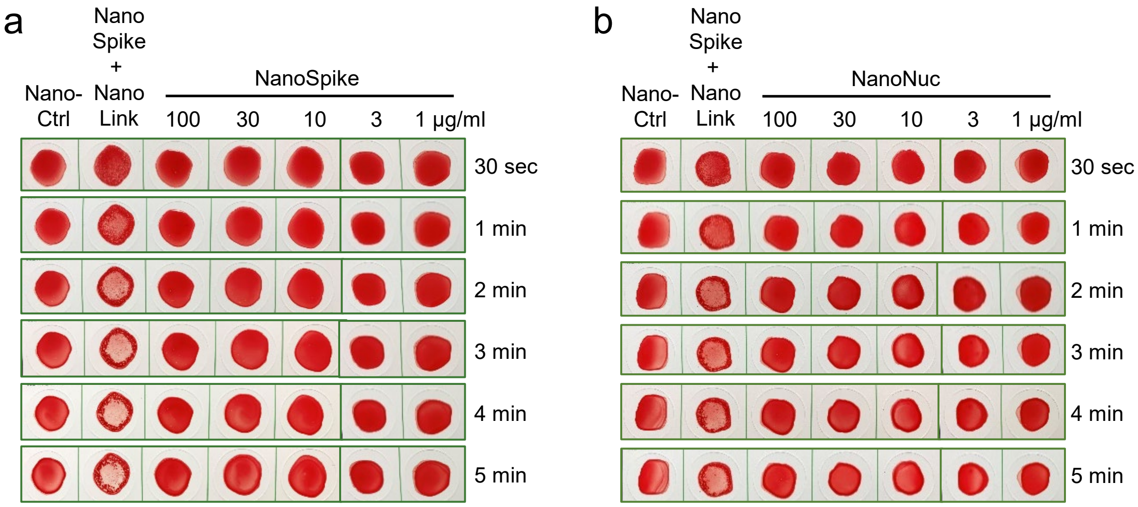

Supplementary Figure 1 Lack of agglutinating function of NanoSpike and NanoNuc in the absence of SARS-CoV-2-specfic AB. (**a,b**) Hemagglutination assay of whole EDTA blood from a COVD19-negative donor that was treated with NanoSpike (a) or NanoNuc (b) at indicated concentrations. Blood treated with NanoControl or a combination of NanoSpike and NanoLink is shown for comparison.

Supplementary Figure 2

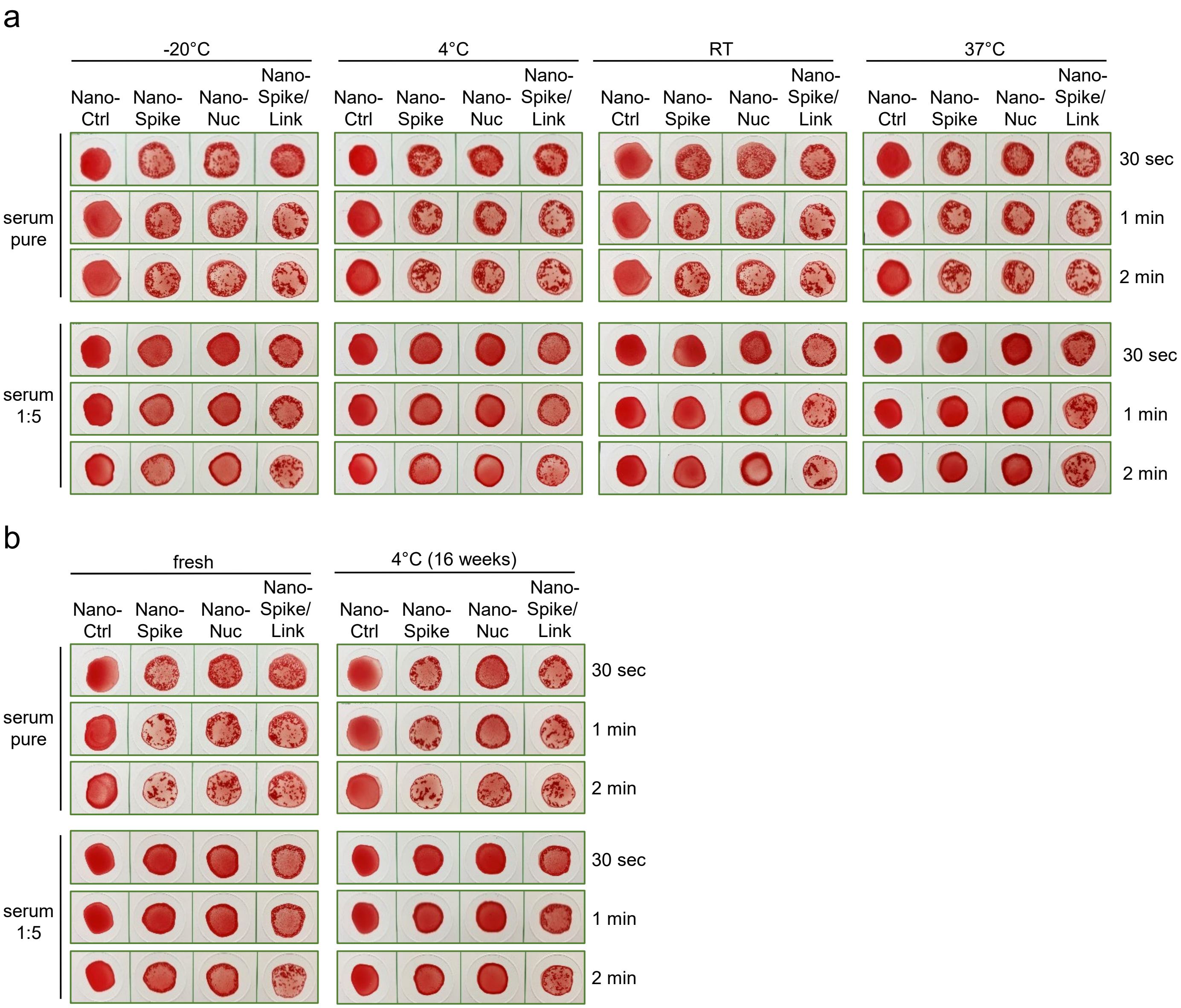

Supplementary Figure 2 Stability of recombinant proteins used in the NanoSpot assay. (**a,b**) Hemagglutination assay based on serum samples (undiluted or 1:5-diluted) from a convalescent COVID-19-patient. The recombinant proteins were stored at the indicated temperatures for four weeks (a) or 16 weeks (b). Proteins stored at -20ºC (a) or freshly prepared proteins (b) served for comparison.

Supplementary Figure 3

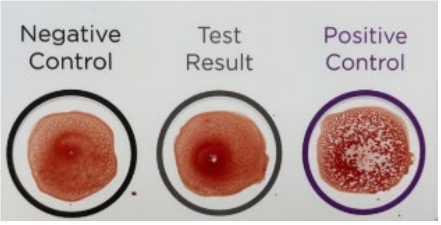

Supplementary Figure 3 Example of low-level spontaneous hemagglutination. Hemagglutination assay of blood from a COVD19-negative donor that was treated with NanoControl (Negative Control), NanoSpike (Test Result) and NanoLink (Positive Control) for one minute. Note the subtle, but clearly visible, agglutination in Negative Control and Test Result samples.

Supplementary Figure 4

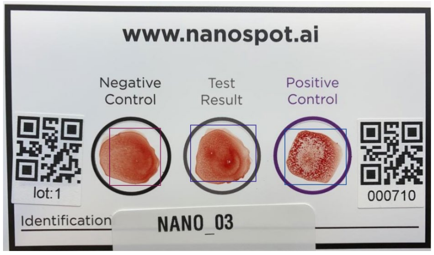

Nano_03
Insufficient focus

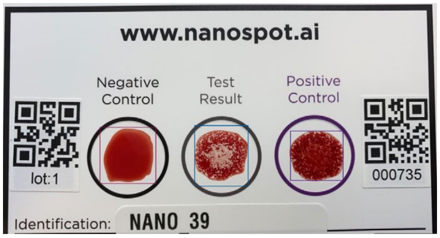

Nano_39
Low confidence score Positive Control (0.29)

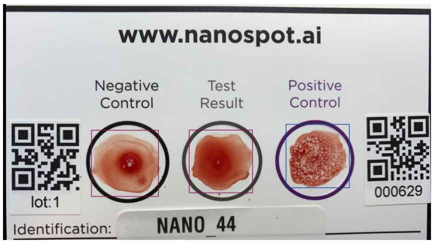

Nano_44
Low confidence score Negative Control (0.36)

Supplementary Figure 4 NanoSpot.ai tests excluded from study due to technical issues

One test with insufficient image quality (blurred focus, Nano_03) and two samples that failed the confidence score of 0.5 (Nano_39, Nano_44) were excluded from test interpretation. Please note that these issues can likely be addressed by improvement of the mobile app (image focusing) and additional training of AI (recognizing artifacts, such as insufficient sample dilution (Nano_39) and air bubbles (Nano_44)).

Supplementary Figure 5

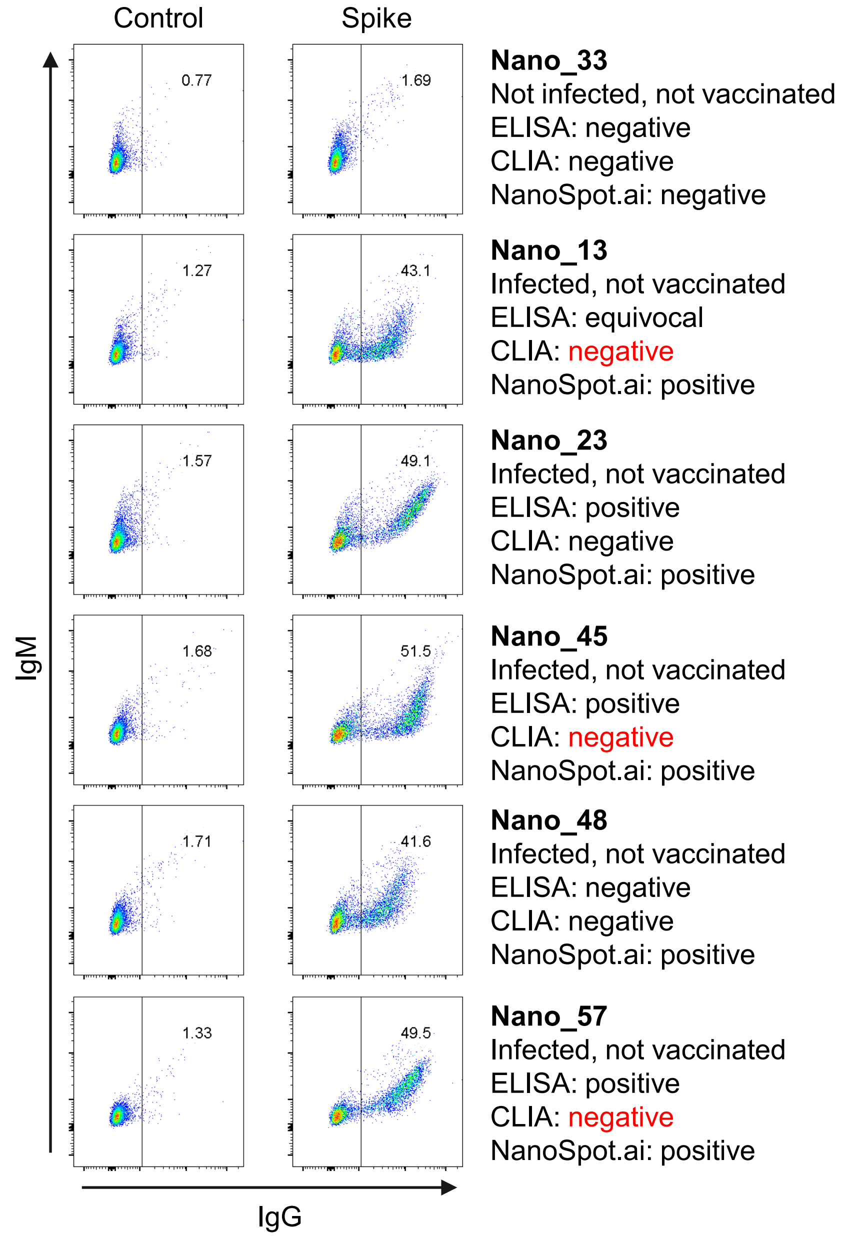

Supplementary Figure 5 S-Flow assay using samples from clinical study

HEK293T cells, which were transfected with a control vector or a SARS-CoV2 Spike expression vector, were incubated with serum from indicated patients, and the presence of αSpike AB was detected by flow cytometry using secondary AB against IgM and IgG, as indicated. Live cells as identified by forward and side scatter are shown.

Supplementary Figure 6

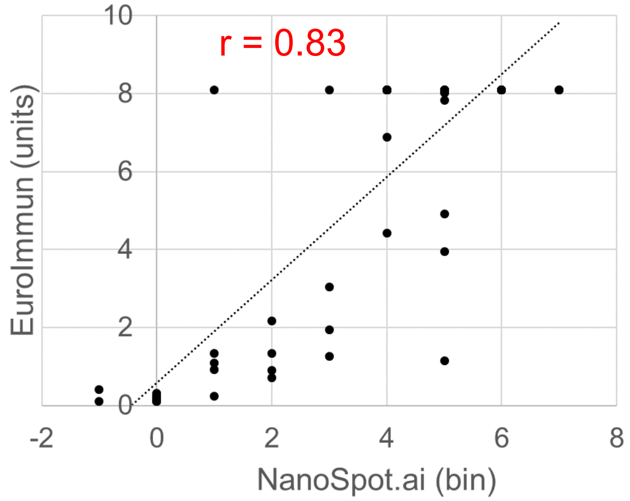

Supplementary Figure 6 Correlation of ELISA and NanoSpot.ai

Results from the clinical study obtained by ELISA (arbitrary units) and NanoSpot.ai (AI bins) were correlated and a trendline was added in Excel. The resulting Pearson correlation index (r), as determined by Excel, is indicated. AI bins obtained by NanoControl were subtracted from AI bins obtained by NanoSpike to eliminate background agglutination.
